# The Effects of Cyclic Loading and Motion on the Implant–Cement Interface and Cement Mantle of PEEK and Cobalt–Chromium Femoral Total Knee Arthroplasty Implants: A Preliminary Study

**DOI:** 10.3390/ma13153323

**Published:** 2020-07-26

**Authors:** Lennert de Ruiter, Raelene M. Cowie, Louise M. Jennings, Adam Briscoe, Dennis Janssen, Nico Verdonschot

**Affiliations:** 1 Orthopaedic Research Laboratory, Radboud university medical center, Radboud Institute for Health Sciences, P.O. Box 9101, 6500 HB Nijmegen, The Netherlands; lennert.deruiter@radboudumc.nl (L.d.R.); Nico.Verdonschot@radboudumc.nl (N.V.); 2 Institute of Medical and Biological Engineering, School of Mechanical Engineering, University of Leeds, Leeds LS2 9JT, UK; r.cowie@leeds.ac.uk (R.M.C.); l.m.jennings@leeds.ac.uk (L.M.J.); 3 Invibio Ltd., Thornton Cleveleys FY5 4QD, UK; a.briscoe@invibio.com; 4 Laboratory of Biomechanical Engineering, University of Twente, P.O. Box 217, 7500 AE Enschede, The Netherlands

**Keywords:** total knee arthroplasty, polyetheretherketone, fixation, debonding, implant–cement interface, PMMA

## Abstract

This study investigated the fixation of a cemented PEEK femoral TKA component. PEEK and CoCr implants were subjected to a walking gait cycle for 10 million cycles (MC), 100,000 cycles or 0 cycles (unloaded control). A method was developed to assess the fixation at the cement–implant interface, which exposed the implants to a fluorescent penetrant dye solution. The lateral condyles of the implants were then sectioned and viewed under fluorescence to investigate bonding at the cement–implant interface and cracking of the cement mantle. When tested for 100,000 cycles, debonding of the cement–implant interface occurred in both PEEK (61%) and CoCr (13%) implants. When the duration of testing was extended (10 MC), the percentage debonding was further increased for both materials to 88% and 61% for PEEK and CoCr, respectively. The unloaded PEEK specimens were 79% debonded, which suggests that, when PEEK femoral components are cemented, complete bonding may never occur. Analysis of cracks in the cement mantle showed an absence of full-thickness cracks in the unloaded control group. For the 100,000-cycle samples, on average, 1.3 and 0.7 cracks were observed for PEEK and CoCr specimens, respectively. After 10 MC, these increased to 24 for PEEK and 19 for CoCr. This was a preliminary study with a limited number of samples investigated, but shows that, after 10 MC under a walking gait, substantial debonding was visible for both PEEK and CoCr implants at the cement–implant interface and no significant difference in the number of cement cracks was found between the two materials.

## 1. Introduction

In cemented total knee arthroplasty (TKA), fixation is achieved by mechanical interlock of the implant with the bone via a layer of polymethylmethacrylate (PMMA) bone cement. During surgery, the doughy cement is typically applied to bone and/or implant surfaces after which the implant is pushed into place. The result is an implant that is well-fixed to the underlying structure, which clinically has demonstrated good long-term survival [1,2,3,4]. To ensure adequate long-term fixation, the implant–cement interface can be strengthened by a number of options. Femoral components are usually designed with cement pockets for macro-interlock, and a surface texture is added to enhance the fixation strength through micro-interlock. Standard application of these features has amounted to decades of evidence of firm and reliable fixation [1,2,3,4]. Additional efforts have been made in the past to further enhance implant fixation by making the material adhere to the cement via chemical bonds rather than just shape-match at a macro- and microscale [5,6,7,8,9]. However, technologies such as PMMA or silane pre-coatings in cemented arthroplasty have not been largely adopted in conventional implant designs, at least in part due to the absence of evidence on clinical efficacy [10,11]. Hence, most femoral TKA implants on the market rely on mechanical interlock of the implant to the cement and consequent fixation of the cement to the bone [12,13,14,15].

**Figure 1:**
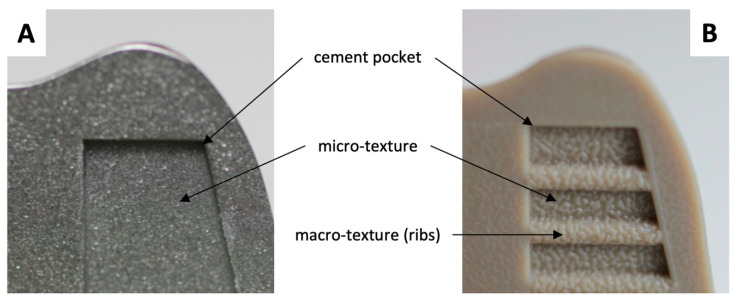
Surface texture of the CoCr (**A**) and PEEK (**B**) implants. The additional cement-bonding features of the PEEK implant are visible, comprising a macroscopic rib features within the cement pockets and a microscopic pattern superimposed over the surface. Adapted from de Ruiter et al. (2017) [26].

For conventional femoral component materials (cobalt chrome, CoCr), a micro-texture is often applied to the fixation surface (Figure 1), and, with this modification, there have been few clinical reports of debonding at the cement–implant interface [16,17,18,19,20,21]. An increasing interest in the investigation of different materials and manufacturing techniques for joint replacements however brings about the potential for different failure modes of the implant. PEEK-OPTIMA™, for example, has been considered as an alternative to CoCr in the femoral component of a TKA to give a metal-free implant [22,23,24,25,26,27,28]. The lower modulus of a PEEK implant compared to CoCr may help to reduce stress shielding but may also change the distribution of forces at the cement–implant interface, which may influence implant fixation. There are potential advantages of investigating different implant materials. With PEEK, for example, the injection molding process used in manufacturing can apply macro- and micro-textures to the fixation surfaces in a one-stage manufacturing technique (Figure 1). A previous study into fixation strength of a PEEK implant with modifications of the fixation surface including the addition of macro- and micro-textures demonstrated an altered distribution of forces at the cement–implant interface compared to CoCr implants. Despite a decrease in fixation strength of PEEK femoral components, the failure modes of the different implant materials were similar, and it was concluded that the bond between implant and cement may be sufficiently strong for clinical use [26].

The fixation at the cement–implant interface is understudied. However, debonding of the femoral component may lead to gross implant loosening, abrasion at the cement–implant interface and failure of the cement mantle. Previous mechanical testing indicated that gross loosening of a PEEK femoral component is very unlikely if the fixation surface has been optimized to provide sufficient mechanical interlock [26]. However, micromotions of a loosened implant could cause abrasion of the cement–implant interface leading to the subsequent release of PEEK and/or cement particles, which may accelerate wear debris induced osteolysis and/or lead to failure of the cement mantle and could produce cement particles leading to third-body wear [29,30]. These potential complications underline the importance of understanding the implant–cement interface, particularly for a PEEK femoral TKA component.

The aim of this study was to determine the quality of the implant–cement interface of a PEEK femoral TKA component and compare it to a CoCr implant. Implants were subjected to clinically relevant loading and motions for up to 10 million cycles (MC) in a knee simulator and a method was developed to assess the bonding between the implant and cement and the integrity of the cement mantle. It was hypothesized that, due to the difference in thermal conductivity and modulus of the implant materials, the bonding at the cement–implant interface and the cracking of the cement would differ between implant materials and that PEEK would show more debonding and cracks in the cement mantle than CoCr. This was a preliminary study to establish a method to evaluate the implant–cement interface and as such was carried out with a small sample size.

## 2. Materials and Methods

### 2.1. Materials

Mid-size (size C) injection molded PEEK-OPTIMA™ femoral components (collaboration partners Maxx Orthopedics Inc., Plymouth Meeting, PA, USA and Invibio Knee Ltd., Thornton-Cleveleys, UK) and MAXX freedom knee (CoCr) femoral components (Maxx Orthopedics Inc., Plymouth Meeting, PA, USA) were used in this study. The implants had a similar geometry, although the macro-features and texture on the fixation surface differed (Figure 1) and was optimized for each femoral component material. The samples were cemented to custom made polyoxymethylene (Delrin^®^) fixtures using Palacos R&G cement (Heraeus, Hanau, Germany). Delrin was chosen as the substrate due to having mechanical properties suitable for 10 MC wear simulation and a low porosity as the testing was carried out in various liquids. While the machined surface of the Delrin fixture may not be clinically relevant, the fixtures were consistent for all samples and the fixture–implant interface was not of interest in this study. The geometry of the Delrin substrate was designed using CAD and based on the geometry of the implant, allowing for a 1-mm cement mantle. The subsequent CAD model was CNC-machined using a five-axis machine. The cement used was the same as that used clinically. It was mixed manually, and the doughy cement was applied to the implant in excess. The implant was pressurized onto the Delrin fixture and shims were used to create a cement mantle with a consistent 1-mm thickness. The process was carried out at room temperature with the same technique applied irrespective of the implant material.

When tested under physiological loading and motion, the femoral components were coupled with Size C all-polyethylene tibial components (Maxx Orthopedics Inc., Mahwah, NY, USA). To assess the debonding of the femoral component–cement interface, the implants were immersed and/or tested in a fluorescent penetrant dye (WB200, Sherwin Babbco) in saline solution at 1:10 concentration.

### 2.2. Experimental Design

**Figure 2:**
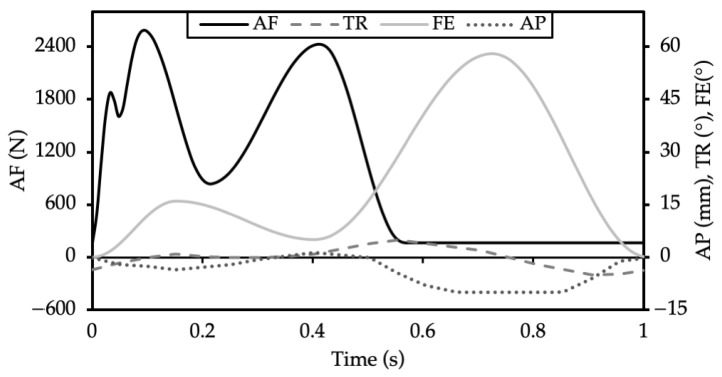
Input profiles for cyclic loading on the knee simulator. The parameters are axial force (AF), tibial rotation (TR), flexion-extension angle (FE) and anterior-posterior displacement (AP).

**Figure 3:**
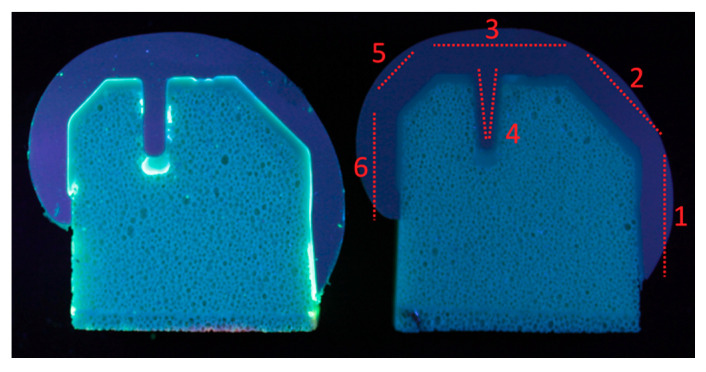
Proof-of-concept lateral condyle PEEK specimens cemented on bone-analog foam after 100,000 cycles in fluorescent dye: (Left) the implant under UV lighting showing complete interface dye fluorescence (score 3); and (Right) the same sample without UV lighting and how the interface was divided into 6 regions for analysis (**1**, anterior flange; **2**, anterior chamfer; **3**, distal area; **4**, peg; **5**, posterior chamfer; **6**, posterior flange).

Three experimental groups with three samples in each were defined, as shown in Table 1. Group 1 components were soaked in the penetrant dye for 27.8 h (equivalent to the duration of 100,000 gait cycles carried out at 1 Hz). This was carried out for PEEK implants only, to assess the initial bonding between the cement and implant (“unloaded control”). Only PEEK implants were investigated in this experimental group because a previous in-house cadaveric experiment demonstrated poor initial fixation for PEEK specimens and good initial fixation between the implant and cement for CoCr components. It was assumed that loading of the CoCr device was required to disrupt the fixation at the cement–implant interface and to encourage the dye into the interface. Group 2 (“Gait control”) comprised both PEEK and CoCr femoral components, which, following cementing onto fixtures, underwent physiological gait loading and motion (Figure 2) in the penetrant dye for 100,000 cycles at 1 Hz (27.8 h) using a six-station ProSim electropneumatic knee simulator (Simulation Solutions, Stockport, UK). The simulator has six degrees of freedom, and four axes of motion were controlled during the test: Axial Force (AF), Flexion Extension (FE), Tibial Rotation (TR) and Anterior Posterior displacement (AP). Performing 100,000 cycles facilitated dye uptake in debonded or cracked areas. To determine the number of gait cycles required for the dye to enter the cement–implant interface, an experiment was carried out in which PEEK implants were cemented to a bone-analog foam and loaded uniaxially (260–2600 N at a frequency of 1 Hz) in the fluorescent dye for 100,000 cycles. At the conclusion of this preliminary study (Figure 3), for PEEK implants, a fluorescent line could be seen between the cement and implant where the dye had entered the interface. It was assumed that dye uptake would be higher when the sample was loaded in a simulator and subjected to simultaneous loading and motion rather than the uniaxial loading used during method development. It was not feasible to use a bone-analog foam as the substrate for the 10 MC simulation, thus, to maintain consistency between samples, Delrin was used as the substrate throughout. Group 3 comprised PEEK and CoCr femoral components that had been previously tested for 10 MC in a ProSim knee simulator under physiological loading and motion to represent the kinetics and kinematics at the bearing surface of the tibiofemoral joint during a walking gait cycle. High flexion activities and forces at the patellofemoral joint were not considered in this study. The experimental wear simulation study was carried out under “Leeds high kinematic” conditions (Figure 2) in a lubricant of 25% bovine serum supplemented with 0.03% sodium azide solution against all-polyethylene tibial components [31]. These test conditions were similar to those previously described by Cowie et al. (2016) [23]. Following wear simulation, the samples were cleaned using detergent ensuring the PEEK and CoCr implants were treated the same in side-by-side studies. This test group (“10 MC gait”) subsequently underwent a further 100,000 cycles under the same loading and motion while immersed in the penetrant dye. All groups were thus exposed to the dye solution for the same duration. 

### 2.3. Analysis of Dye Penetration (Fluorescence)

Having been immersed in the dye, the specimens were sectioned in the sagittal plane through the center of the lateral condyle with a cutting blade under water cooling. The lateral condyle was chosen over the medial condyle because, when cross-sectioned, all internal implant faces are visible (Figure 3); for the medial condyle, the geometry of the implant means that when cross-sectioned through the center of the condyle, the anterior chamfer cannot be seen. A UV light was used to excite the fluorescent dye (320–420 μm) and imaging performed using a generic microscope at 1.5 × 10 magnification. A scoring system was devised to assess whether fluorescent dye was visible at the implant–cement interface. The femoral components were divided into six distinct regions for analysis (Figure 3): the anterior flange, the anterior chamfer, the distal area, the peg, the posterior chamfer and the posterior flange. No differentiation was made for the intensity of the UV-light since debonding was assumed to be complete in regions where fluorescence was observed regardless of the light intensity. Scoring was carried out manually by two scorers and each area was ranked between 0 and 3 (0: no fluorescence; 1: up to 33% of the interface fluorescent; 2: up to 67% of the interface fluorescent; and 3: complete fluorescence). There was a high level of inter-observer reliability resulting in few discrepancies between the two scorers. When differences were identified, researchers deliberated and agreed on a final score. Separate area scores were summed for the entire interface and averaged to obtain a single score for each specimen.

### 2.4. Analysis of Cement Damage (Full-Thickness Cracks)

Cement damage was scored for each of the six regions on the femoral component described in Figure 3 by assessing the number and location of cracks that crossed the full thickness of the cement mantle. Again, two scorers independently examined the implants. Few differences between scorers were identified and were reviewed and debated until a consensus was reached. The dataset was checked and corrected for double hits in overlapping images. The number of cracks in each region was averaged for all specimens in the study group. Average scores were compared between PEEK and CoCr, for both the entire cement mantle and each separate region.

### 2.5. Statistics

The data are presented as the mean (±standard deviation) for both fluorescence and full-thickness cracks. Statistical analysis was carried out in SPSS 24.0 (IBM Corp, Armonk, NY, USA) using a t-test to compare PEEK and CoCr for each experimental group, under the hypothesis that PEEK would show more fluorescence and cracks than CoCr. Groups were analyzed with a 0.05 significance level.

## 3. Results

### 3.1. Analysis of Dye Penetration (Fluorescence)

**Figure 4:**
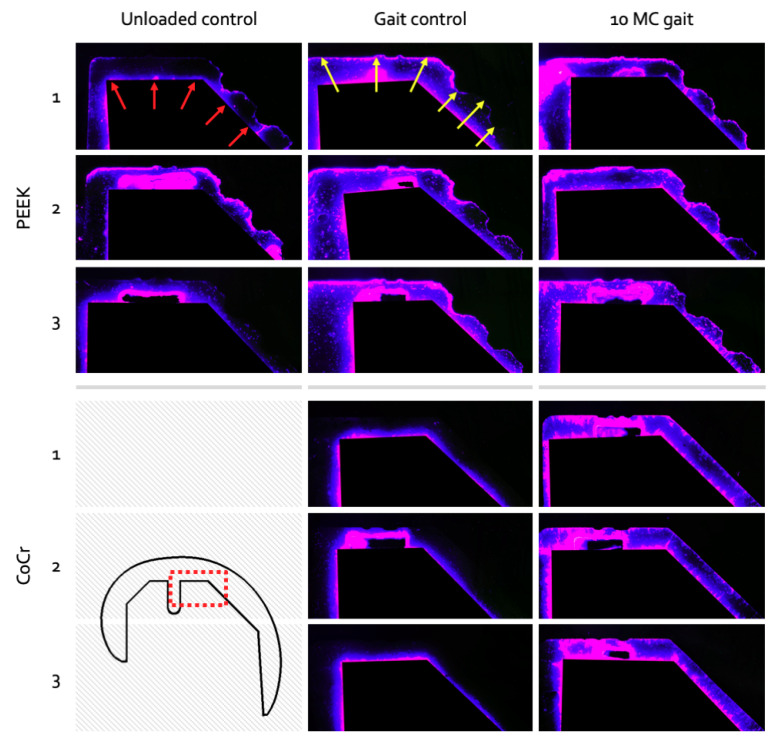
Test specimens under UV lighting show dye fluorescence intensity as blue-to-pink coloration with the pink area highlighting the highest dye uptake. Both cement–Delrin (red arrows) and implant–cement (yellow arrows) interfaces are visible. A section of the anterodistal cement mantle is shown at the three time intervals to demonstrate the appearance of dye penetration in both the PEEK and CoCr group. The variability within the PEEK soaked control group is noticeable, ranging from near-full bonding (Specimen 3) to complete debonding (Specimen 2). A clear evolution of dye penetrance in the CoCr can be seen, where no implant–cement interface is visible in the loaded controls, but full interface fluorescent is visible in the Group 3 samples.

**Figure 5:**
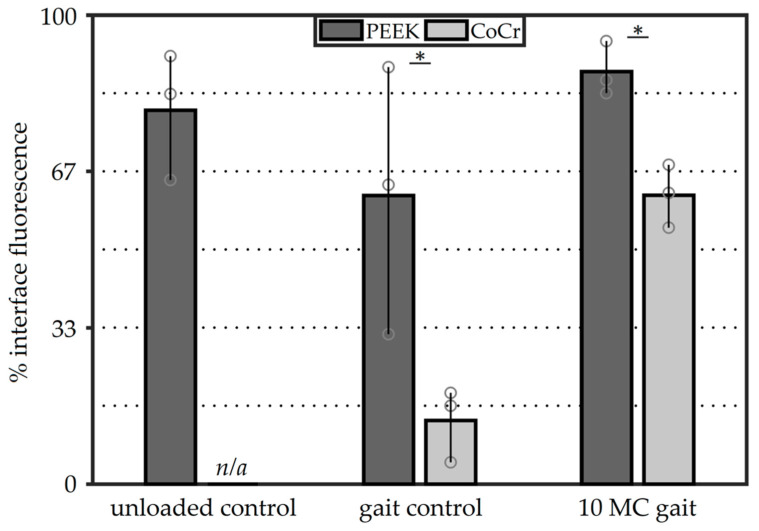
Average interface fluorescence scores of the complete implant–cement interfaces at difference intervals. The bars show the range of observations with the individual specimens shown as circles (statistical analysis compares PEEK to CoCr; * denotes *p* < 0.05).

Unloaded PEEK control specimens (Group 1) showed high levels of dye penetration at the implant–cement interface without the components undergoing loading and motion (Figure 4). On average, 79% (±11%) of the PEEK–cement interface was fluorescent after being soaked in dye (unloaded) for 28 h (Figure 5). After 100,000 gait cycles, for the PEEK gait controls (Group 2), the average fluorescence area was 61% (±23%). This was lower than the unloaded controls (Group 1), but with a larger variability between samples. The CoCr Group 2 gait control samples showed limited dye penetration at the interface (13% (±6%)) after 100,000 gait cycles (Figure 4). After an extended number (10 MC) of test cycles under physiological loading and motion (Group 3), the implant–cement interfaces were easily distinguishable for both femoral component materials (Figure 4). For Groups 2 and 3, the PEEK components showed significantly (*p* < 0.05) more fluorescence than the CoCr implants. Comparing the gait controls (Group 2) to the implants loaded for an extended number of cycles (Group 3), the PEEK femoral components showed a slight increase in percentage fluorescence, from 61% (±23%) to 88% (±5%), while the CoCr implants displayed a steep increase after 10 MC of simulation, from 13% (±6%) to 62% (±6%) interface fluorescence (Figure 5). The variability between the samples for the Group 3 implants was lower than the other groups for both material types. 

### 3.2. Analysis of Cement Damage

**Figure 6:**
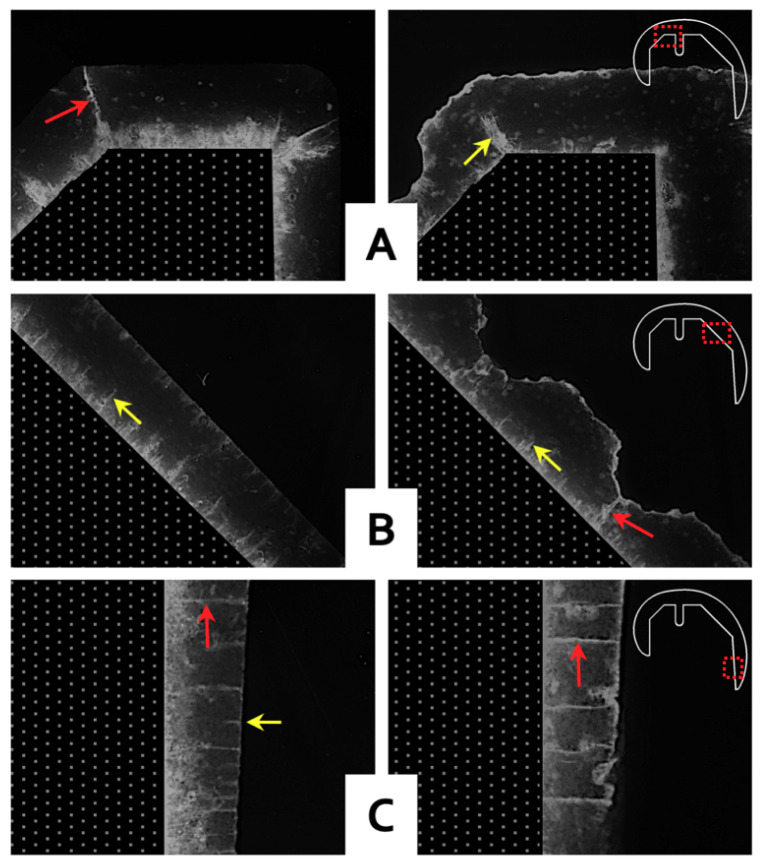
Representative images of CoCr (left) and PEEK (right) reconstructions after 10 MC, showing dye penetration in cement mantle cracks at three locations (**A**–**C**) around the femoral component as indicated by the red squares in the detail-figures. The red arrows indicate full-thickness cracks in the PMMA cement, the yellow arrows incomplete cracks. The reduced cement mantle thickness, caused by the ridges in the PEEK surface, is visible in (**B**) (right).

Full-thickness cracks were observed in the cement mantles against both the PEEK and CoCr femoral components for the gait controls and 10 MC test groups. The locations of these cracks however were markedly different with cracks at the interface chamfers more often observed with a CoCr component than with a PEEK implant. With CoCr, the cracks tended to run the full thickness of the cement mantle, as opposed to with PEEK where the cracks at the chamfers were mostly incomplete (Figure 6A). In the PEEK femoral components, at the apex of the ridges which were incorporated into the cement pockets to enhance fixation, full-thickness cracks were common in the Group 3 femoral components which had been previously tested for 10 MC (Figure 6B). Both CoCr and PEEK reconstructions showed similar crack patterns in the anterior and posterior flange areas. In this region, the cracks underneath the PEEK components generally resulted in full-thickness cracks, while those underneath the CoCr implants showed both full-thickness cracks and numerous small cracks (Figure 6C).

**Figure 7:**
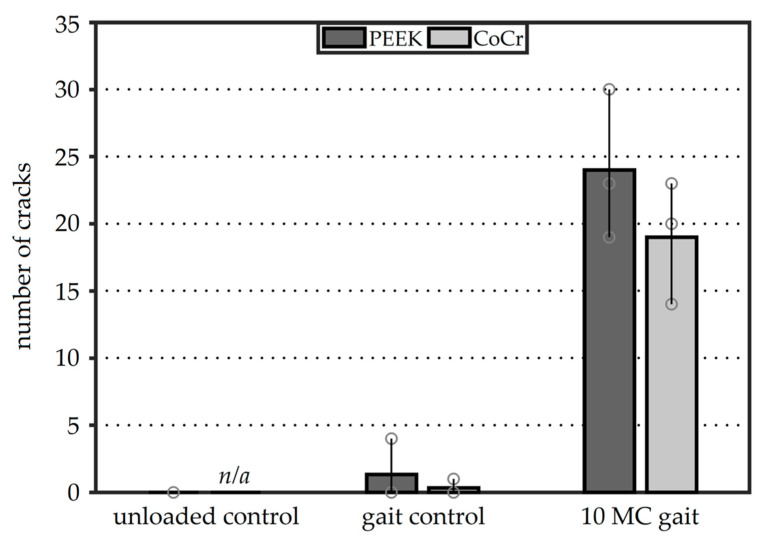
The average number of full-thickness cracks for the three experimental groups. The bars show the range of observations with the individual specimens shown as circles.

No full-thickness cracks were observed in the unloaded control PEEK femoral components (Figure 7). In the gait control femoral components, some full-thickness cracks appeared: 1.3 (±1.9) and 0.7 (±0.9) cracks on average for PEEK and CoCr, respectively. This difference however was not significant (*p* > 0.05). After 10 MC under gait conditions, the number of cracks in both reconstructions had substantially increased. The average number of cracks in the cement layer below the PEEK femoral components was 24 (±4.5), while the CoCr reconstructions demonstrated a mean of 19 (±3.7) full-thickness cracks. Again, this difference was not significant (*p* > 0.05).

**Figure 8:**
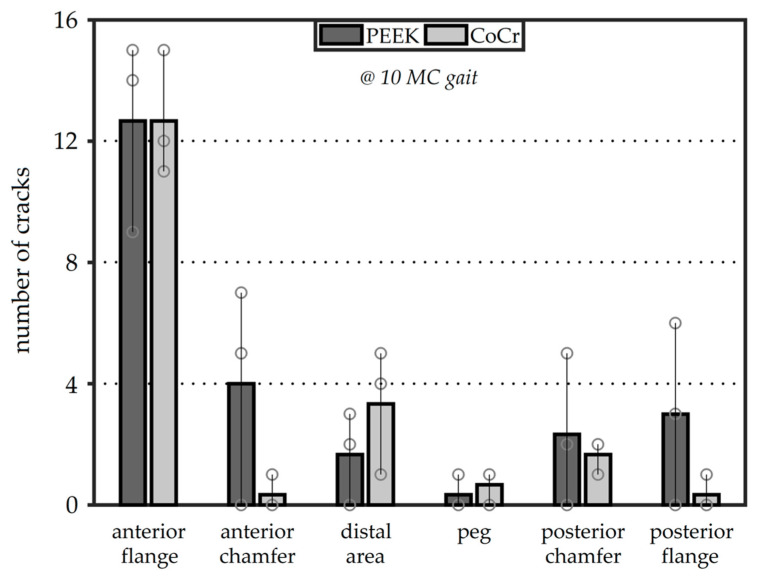
The average number of full-thickness cracks separated for each region after 10 MC. The bars show the range of observations with the individual specimens shown as circles.

Further examination of the crack locations after 10 MC revealed that most cracks occurred in the cement mantle below the anterior flange of the implant (Figure 8). For both femoral component materials, the average number of cracks was 12.7 in this region. For the CoCr components, few cracks were visible around the anterior chamfer, while the PEEK implants showed cracks in the cement in this area at the apex of the fixation ridges, as shown in Figure 6B. The posterior flange cement area also showed more cracking with the PEEK implant than with CoCr. After 10 MC, the femoral component material did not influence the number of cracks in the distal area, peg or posterior chamfer regions (*p* > 0.05).

## 4. Discussion

The aim of this study was to assess and compare the femoral component cement–implant interface for TKAs manufactured from PEEK and CoCr. The two femoral component materials were shown to have different effects on the cement mantle due to differences in the material properties of the components and variations in both the geometry and topography of the fixation surface. In summary, the CoCr implants showed less dye penetration at the cement–implant interface than PEEK components, indicating superior adhesion between CoCr and cement. However, debonding of the cement–implant interface was evident for all implants when tested for high numbers of cycles. The integrity of the cement mantle was also analyzed. Cracks were evident in the cement beneath both PEEK and CoCr femoral components. There was no significant difference between the number of cracks in the cement–CoCr or cement–PEEK interface, but the location of the cracks differed depending on the implant material.

### 4.1. Analysis of Dye Penetration

The PEEK unloaded soaked control specimens showed that PMMA cement did not fully bond to the PEEK implant. Dye penetration did not differ between unloaded (Group 1) PEEK femoral components and those tested for 10 MC, (Group 3) specimens. Therefore, 10 MC experimental simulation had no additional effect on the bonding state of the PEEK implant–cement interface and in no samples was gross implant loosening observed. A previous pull-off fixation study by De Ruiter et al. (2017) concluded that PEEK femoral components with the same profile on the fixation surface as used in this study have adequate fixation strength [26]. The information from these two studies combined suggests that debonding of the PEEK–cement interface does not necessarily limit the long-term mechanical fixation of the construct as a whole, although, in both studies, non-physiological bone surrogates were used. This is further emphasized by the fact that substantial interface debonding was seen with CoCr implants following testing for 10 MC. CoCr femoral components are known to be mechanically stable when implanted for periods in excess of 10 years. Clinical evidence from successful polyethylene (PE) implants show similar debonding scenarios. All-polymer PE tibial TKA components, for example, have been available for several decades and have very positive outcomes, despite PE having no intrinsic bond with the cement [32,33,34]. Similarly, cemented all-polymer PE acetabular cups for hip arthroplasty, which are dependent on surface textures for fixation, also show excellent survival rates [1,3,35], and a clinical trial of a Delrin femoral component showed a low incidence of loosening after 10 years implantation [36]. 

The cause of the immediate PEEK–cement interface debonding can be attributed to the lack of adhesion between the cement and implant, which means fixation primarily relies on mechanical macro- and micro-interlock with the surface topographical features on the implant fixation surface. The poorer thermal conductivity of PEEK compared to CoCr may reduce the dissipation of heat produced as the PMMA cement cures which may lead to shrinkage of the cement contributing to debonding of the PEEK–cement interface. In addition, in clinical practice, the bond may be further influenced by contamination of the interface by blood or fat, as well as poor cementing technique or timing. From that perspective, the clean surfaces and absence of time pressure under which the samples were prepared for this study represent the optimal conditions for obtaining a well-fixed implant–cement interface.

### 4.2. Analysis of Cement Cracks

Macroscopic damage evaluation showed that full thickness cracks were present in both CoCr and PEEK reconstructions tested under a gait cycle. Following 10 MC gait simulation, the mean number of full-thickness cracks in the cement mantle was approximately 20% higher for PEEK implants compared to CoCr; however, this difference was not significant (*p* > 0.05). With the addition of potential stress risers (ridges in the fixation surface), which lead to a thinner cement mantle at the ridge-locations, the difference between the PEEK and CoCr components was expected to be larger. However, perhaps with the CoCr component, a different failure mechanism occurred. It is postulated that the higher stiffness of CoCr compared to PEEK also gives the potential for higher local stresses in the cement–CoCr implant interface as the femoral component is less compliant. Previous studies have shown the stiff CoCr component to generate high stress peaks in the cement underneath the proximal anterior flange [24,25,26,37]. Many small cracks were visible in this region. Analysis of these small cracks was beyond the scope of this study but there is potential for these small cracks to grow, which may further increase cement damage in the anterior flange [20,24,25]. The numbers of cracks in cement at the anterior flange and posterior chamfer areas of the cement mantle were similar in both implants; however, there was a greater number of cracks in the PEEK–cement interface in the anterior chamfer and the posterior flange compared to the CoCr–cement interface. 

### 4.3. Limitations

There are a number of limitations associated with this study which should be considered in the clinical interpretation of these findings. Firstly, the study was performed on an experimental wear simulator. As such, it is designed to mimic the kinetics and kinematics at the implant bearing surface as opposed to the femoral/fixture (bone) interface. The femoral components were cemented onto custom-shaped Delrin blocks that were mounted into the simulator. This replaced the in-vivo cement–bone interface with a cement–Delrin interface. It is acknowledged that Delrin may not represent bone in terms of its porosity, elastic modulus and surface texture. However, for this study, which involved extensive wear simulation (>6 months) in a biological lubricant applying forces up to 2.8 kN, it was considered appropriate and was easy to section to allow analysis of the cement–implant interface. The intention was not to study the Delrin–cement interface and, therefore, the surface was consistent (as machined) for all experimental groups, and, other than pockets to accommodate the pegs, no additional features were introduced into the surface of the Delrin, which provided a shape-lock fixation. The different mechanical properties of Delrin compared to bone may however have changed the load distribution in the cement mantle and how this influenced interface debonding and cement mantle damage is unknown. The 10 MC of gait simulation, equivalent to the loading the implant undergoes following approximately 10 years use in a moderately active patient [38], assumes that, throughout the duration of implantation, the implant remains fully supported with no resorption of the underlying bone. It is not known whether the relatively sharp corners at the chamfers of the Delrin block may have stimulated crack propagation or whether the smooth Delrin surface may have reduced stress risers created by individual trabeculae, which may play an important role in crack initiation [39]. Hence, the exact effect of the use of Delrin is unclear and future studies could consider a more physiologically relevant substrate. In terms of the cementing of the implants, there were further limitations as it was prepared using a manual mixing technique, which is inferior to the vacuum mixing process routinely used in the clinic. Manual mixing of cement gives rise to the potential for entrapment of air during cement preparation, which may cause pores. During analysis of the cement mantles, however, no pores were found in the cement layer, which may be attributed to the well-controlled laboratory conditions in which the reconstructions were prepared.

In addition, the simulator included only tibiofemoral contact. When both the tibiofemoral and patellofemoral joints are considered, in vivo forces are higher, since patellofemoral contact is a major contributor to the total loads on the femoral component particularly at higher flexion angles [24]. However, the increased forces due to patellofemoral contact do not necessarily lead to an increased risk of debonding. A study by Berahmani et al. (2016) into micromotions behind cementless femoral knee components concluded that the patellofemoral contact decreased the micromotions in the anterior flange up to 22% [40]. The study also investigated a gait cycle only. Although high-flexion activities are carried out less frequently than level gait, more strenuous activities including stair climbing, standing up and squatting may influence the study findings; however, the comparative nature of this study comparing the cement–implant interface of PEEK to that of CoCr is a strength.

Furthermore, only three samples were investigated in each experimental group; this was limited by the extended duration of the studies within excess of six months continual testing required to prepare the 10-MC gait samples. Future work should consider larger implant sizes and perhaps extending the number of timepoints investigated especially for the CoCr implants to gain a better understanding of when debonding of these implants occurs and including a CoCr Group 1 investigation to better understand the initial fixation of CoCr implants. However, increasing the sample size may necessitate automation of the analysis protocols to minimize variability between samples. The protocol used merely considers the loading the implants undergo but not whether degeneration of the cement occurs during ageing. Further method development would be required to understand this and whether the mechanical properties of cement change with time. To minimize errors between experimental groups associated with ageing effects, the PEEK and CoCr implants were tested in parallel.

Finally, the study outcome parameters, dye penetration and cement cracking, are a local 2D representation of the full cement mantle and are thus subject to extrapolation error. The lateral condyle was chosen to represent all areas from anterior to posterior flange which does not cover the entire surface area. However, cracks in this section did propagate further laterally and medially into the cement mantle, and dye visualized at this location must have travelled from external boundaries, which supports the extrapolation applied here. This research did not include a microscopic damage assessment of the implant or cement interface surfaces after 10 MC of experimental simulation. One of the hypothesized results of long-term interface micromotions is the formation of wear particles. Once formed, these could travel into the joint space where they may initiate inflammatory processes which could contribute to wear debris induced osteolysis leading to implant loosening or, if larger particles were to migrate between the articulating surfaces, they could act as a third-body particle and may accelerate bearing surface damage or wear [29,41,42]. 

## 5. Conclusions

This study aimed to develop a method to assess the cement–implant interface bonding and the integrity of the cement mantle. This was done using a fluorescent penetrant dye and was then used to assess CoCr and PEEK femoral components which had undergone up to 10 million walking gait cycles. The study showed poor initial bonding of the PEEK–cement interface; however, after 10 MC simulation, the bonding of the implant remained similar to that of the controls. For CoCr implants, good fixation was measured for the gait control samples, but, after 10 MC, substantial implant–cement interface debonding occurred. After 10 MC, there was no significant difference in implant–cement debonding for the femoral component materials investigated, nor were there significant differences in macroscopic damage of the cement mantle. Further investigations either using a more physiologically relevant simulation system or through either animal studies or a clinical trial may be necessary to confirm these findings.

## Figures and Tables

**Table 1:** The experimental groups and sample size for each femoral component material.

	Group 1	Group 2	Group 3
	Unloaded control	Gait control	10 MC gait
PEEK	3	3	3
CoCr	-	3	3
